# Framework for identifying reference countries in drug safety evaluation: an application of the analytic hierarchy process

**DOI:** 10.1080/20523211.2026.2676817

**Published:** 2026-06-01

**Authors:** Gyeyoung Choi, Seungjin Bae

**Affiliations:** College of Pharmacy, Ewha Womans University, Seoul, South Korea

**Keywords:** Pharmaceutical regulation, overseas safety monitoring, reference countries, focus group interview, Analytic Hierarchy Process

## Abstract

**Background:**

Effective monitoring of global drug safety information is increasingly important for public health. However, the large volume of global safety data, combined with variations in pharmaceutical systems and national regulatory contexts, poses challenges in prioritizing relevant information. To address this challenge, this study aimed to develop a set of criteria for selecting reference countries when evaluating foreign drug safety data.

**Methods:**

Experts from academia and the pharmaceutical industry participated in focus group interviews (FGIs) to identify key factors for selecting reference countries. The Analytic Hierarchy Process (AHP) was then used to determine the relative importance of these factors. Additional FGIs were conducted to assess the validity of the resulting framework, including the relative weights of each criterion.

**Results:**

A total of 11 criteria were identified – six country-specific and five drug-specific. Among the country-specific criteria, ‘countries officially being referenced’ (weight = 0.374) and ‘countries with strong pharmaceutical safety management’ (0.256) were rated as most important. For drug-specific criteria, ‘reliability of the report’ (0.433) and ‘accessibility of the report’ (0.181) were prioritized. In contrast, criteria such as ‘countries with advanced economies’ (0.060) and ‘drug’s country of origin’ (0.090) were considered less significant. Experts from both academia and industry generally supported the prioritization.

**Conclusions:**

This study proposes a structured, evidence-based framework for referencing overseas pharmaceutical safety information. By assigning relative weights to country – and drug-specific criteria, the framework facilitates more consistent and efficient decision-making by national regulatory authorities, particularly in contexts where global safety data are extensive and pharmaceutical regulatory environments vary significantly. Given its basis in a regulatory environment that heavily relies on foreign safety data, the framework may be adapted for use in other countries with similar pharmacovigilance needs.

## Background

The globalization of pharmaceutical trade has made referencing international drug safety information essential for effective post-marketing safety management (European Committee, n.d.). In 2023, the European Union's pharmaceutical trade continued to grow steadily, reaching $277 billion in exports and $119 billion in imports (EUROSTAT, 2024). In Korea, 2,207 drug products were imported in 2022, accounting for approximately 10% of the domestic pharmaceutical market (Health Insurance Review and Assessment Service, 2023). These imports originated from various countries, including the United States, Germany, China, the United Kingdom, Switzerland, Japan, and France, reflecting the global nature of pharmaceutical supply chains.

Given this international context, many regulatory authorities, such as the United States Food and Drug Administration (US FDA) and the European Medicines Agency (EMA), routinely reference foreign safety data when evaluating post-marketing risks. For example, the US FDA collects international safety reports following the International Committee of Harmonization (ICH) E2B guidelines through the FDA Adverse Event Reporting System (FAERS), while the EMA operates its pharmacovigilance (PV) framework, including the Pharmacovigilance Risk Assessment Committee (PRAC) (US FDA, 2024). However, the large volume and heterogeneity of global data pose substantial challenges to timely and accurate risk assessment (Patadia et al., 2024). These challenges are further intensified in low- and middle-income countries, where limited resources and capacity constraints hinder the development and maintenance of robust PV systems (Abiri & Johnson, 2019).

Further complicating global safety monitoring are differences in regulatory frameworks, healthcare systems, pharmaceutical market structures, and patient populations across countries, which contribute to inconsistencies in how safety data are generated, interpreted, and reported (Bhasale et al., 2021). Such inconsistencies complicate PV practices (Garashi et al., 2022; Khan et al., 2023) and underscore the need for context-specific approaches when referencing foreign information, particularly in urgent situations requiring timely and appropriate decision-making.

Although the ICH Good Clinical Practice (GCP) framework provides general guidance, its implementation varies by region, resulting in discrepancies in safety assessments and risk management (Hans & Gupta, 2018). To mitigate these disparities, PV experts proposed recommendations for national regulatory authorities (NRAs) (Peters et al., 2021). Nevertheless, despite ongoing efforts to harmonize international practices, no established framework currently exists to guide the prioritization of foreign safety information. In practice, regulatory authorities and pharmaceutical companies often rely on informal heuristics or prior experiences to determine which countries’ decisions or data to reference (Bujar et al., 2016). This inconsistency may delay or complicate regulatory responses.

To address this gap, the present study proposes a structured framework for referencing overseas pharmaceutical safety information by identifying and ranking key country- and drug-level criteria. By quantifying the relative importance of these factors through expert input, the framework aims to support evidence-based, efficient regulatory decision-making, particularly in situations involving large, heterogeneous, and time-sensitive data sets.

## Methods

### Overview

**Figure 1.**
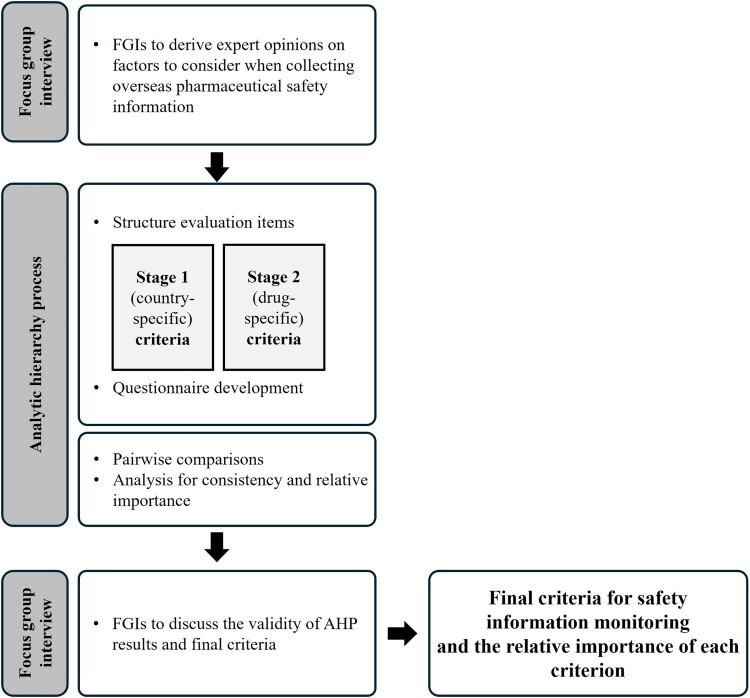
Flowchart of the overall study process.

This study employed focus group interviews (FGIs) and the Analytic Hierarchy Process (AHP) to identify and prioritize criteria for selecting reference countries in the context of international drug safety monitoring. Experts from academia and the pharmaceutical industry participated in the FGIs to identify key criteria. The AHP was subsequently used to assign relative weights to each criterion. To validate the results, a second round of FGIs was conducted. Figure 1 illustrates the overall study process.

### Focus group interviews

The FGIs were conducted for two primary purposes: (1) to gather diverse expert opinions on the criteria for selecting reference countries for pharmaceutical safety information; and (2) to assess the appropriateness of the criteria and the resulting framework, including the relative importance of each criterion, as determined through the AHP.

A total of 14 experts from academia and the pharmaceutical industry participated in the FGIs. The academic group comprised six professors specializing in pharmacoepidemiology, each with over 10 years of experience. The industry group consisted of eight experts recommended by the Korea Research-based Pharma Industry Association (KRPIA), including four professionals specializing in PV and four in regulatory affairs (RA). All industry participants held managerial or executive-level positions and had more than 20 years of experience in the pharmaceutical sector.

The FGIs were conducted in two sequential rounds. The first round focused on identifying preliminary selection criteria for reference countries, addressing both country-specific and drug-specific considerations. The second round, conducted following the AHP-based prioritization, aimed to validate the relevance and robustness of the derived results. Discussions with academic experts concentrated on the domestic and global landscape of pharmaceutical safety information management and international referencing practices. In contrast, industry expert discussions centered on practical considerations, such as drug monitoring standards and real-world applicability.

The focus group interviews (FGIs) were exempt from ethics review in accordance with the Bioethics and Safety Act of the Republic of Korea (Korea Law Information Center, 2023). Further details are provided in the Supplemental Material.

### Analytic hierarchy process

The AHP is a structured method for deriving ratio scales from paired comparisons (Saaty, 1990). It allows for the estimation of relative weights across multiple criteria by systematically incorporating expert judgment. AHP was selected for this study because it is well-suited to decision-making contexts with limited empirical data and modest sample sizes (Saaty, 2016), and allows direct elicitation of criterion weights without requiring predefined performance data. In contrast to other multi-criteria decision analysis methods such as TOPSIS or PROMETHEE, which rely on quantitative performance matrices, and Delphi-based approaches, which lack a formal consistency check, AHP provides a transparent and reproducible framework with an explicit assessment of judgment consistency (Velasquez & Hester, 2013).

The AHP was employed to derive the relative weights of each criterion for selecting reference countries. The analysis included input from 12 academic experts and 16 professionals from the pharmaceutical industry. The process comprised several key steps: identifying levels and elements, defining concepts, formulating comparison questions, and conducting evaluations through paired comparisons (Vargas, 1990).

### Questionnaire development

To ensure clarity and consistency among respondents, the questionnaire presented each criterion alongside a concise definition, as derived from the previously conducted FGIs. Pairwise comparison questions were structured using Saaty’s 9-point scale (Tavana et al., 2023), in which opposing items are placed at opposite ends of the scale, and values 3, 5, 7, 9, along with their reciprocals, are symmetrically distributed around a central value of 1. Table 1 presents the definitions and descriptions corresponding to each level of importance (Saaty, 1990). Participants were instructed to select the value that best represented the criterion they considered more important, thereby indicating its relative weight. A score of 1 denoted equal importance between the two items. (The questionnaire used in this study is available in the Supplemental Material.)

**Table 1.** Fundamental scale of absolute numbers in the Analytic Hierarchy Process.

Intensity of importance	Definition	Explanation
1	Equal importance	Two criteria contribute equally to achieving the objective
3	Moderate importance	Experience and judgment moderately favor one criterion over the other
5	Strong importance	Experience and judgment strongly favor one criterion over the other
7	Very strong importance	Experience and judgment very strongly favor one criterion over the other, but not with certainty
9	Extreme importance	Experience and judgment overwhelmingly support one criterion over the other, with the highest degree of certainty

### Pairwise comparison

The pairwise comparison survey was conducted from September to October 2024, involving 28 participants. This group included 14 experts who had previously contributed to defining the selection criteria during the FGIs. Among the final participants, 12 were from academia, while 16 were from the pharmaceutical industry, evenly divided into two groups: eight from RA and eight from PV. Participants were initially provided with an overview of the survey’s background and objectives, followed by comprehensive explanations of each criterion and the AHP response method. Responses were collected via email.

### Analysis

The consistency ratio (CR) was calculated to assess the consistency of the survey results. Responses were considered valid if the CR was below 0.15 (Saaty, 1983). This threshold was selected to balance the number of expert input with methodological rigor, as CR values up to 0.15 are considered acceptable in AHP studies involving larger or more complex matrices (Baek, 2025; Subramanian & Ramanathan, 2012). Respondent with CR values exceeding this threshold were requested to revise their answers. If the revised responses still failed to meet the threshold, they were excluded from the final analysis. A deterministic aggregation approach was employed by synthesizing Individual judgment matrices through the geometric mean of corresponding entries, assuming equal weight for all respondents (Basak & Saaty, 1993). This approach is commonly used in AHP to preserve the reciprocal structure of pairwise comparisons and to ensure consistency in group judgments. Expertise-weighted aggregation was not applied, as defining objective and reproducible weighting criteria for expert importance was not feasible and could introduce additional bias. The pairwise comparison matrix was structured as a reciprocal matrix with values of 1 along the diagonal. The relative weights were derived using the normalized column method, whereby each element was divided by the sum of its respective column. The normalized values were then averaged across each row to compute the weight of each criterion. A sensitivity analysis was also conducted to assess the robustness of the results using a stricter CR threshold of 0.10. All analyses were conducted using Microsoft Excel^Ⓡ^.

## Results

### Identification of preliminary criteria through FGIs

The interviewees generally agreed that proposing criteria for selecting reference countries to monitor international safety information is a challenging process, but could provide valuable support for establishing a standardized approach to collecting overseas drug safety data.

Table 2 presents the main items identified as possible criteria for inclusion in the AHP, along with the level of consensus reached among participants in each FGI group. A detailed flow diagram illustrating the classification and content-level refinement of candidate criteria is provided in Supplemental Fig. S1.

**Table 2.** Summary of FGI-derived items and expert opinions on inclusion in the framework*.

	Academic group	Industry group
Cross-reference status between countries	O	
The size of the economy or the pharmaceutical industry.	Δ	
Reliability of the country’s PV system	O	Δ
Same indication	O	Δ
Quality of the safety report	O	
Accessibility of the safety report	O	Δ
Demographic similarity of the target population	O	X
Drug’s country of origin	O	X

(O) Participants agreed that the factor is an important consideration for selecting a reference country.

(X) Participants disagreed with the inclusion of the factor and recommended its exclusion.

(Δ) No consensus was reached; participants proposed revising or clarifying the criterion.

*Expert opinions were assessed based on the criterion that 80% or more of participants within each group expressed the same view.

Both groups agreed that cross-reference status between countries should be included (Table 2). The academic group emphasized ‘the extent to which a regulatory body is referred to by other countries and how actively it monitors foreign data,’ whereas the industry group stressed the importance of having ‘an organization that comprehensively collects and evaluates international safety information.’

Regarding the size of the economy or pharmaceutical industry, both groups recommended subdividing the criterion into more specific components (Table 2). Academic experts noted that ‘a large economy does not necessarily imply a large pharmaceutical market,’ suggesting that economic size and the pharmaceutical industry size should be evaluated separately. In contrast, the industry group focused on market size, noting that ‘a large pharmaceutical market likely leads to higher drug sales and, consequently, more accumulated safety data.’

For the reliability of a country’s PV system, the academic group emphasized the importance of ‘a reliable database and reporting system for active surveillance.’ The industry group further noted that ‘ICH membership status and the regulatory agency’s capacity, such as the size of the PV department, may also be relevant.’ (Table 2)

Regarding the drug’s indication in the safety report, the academic group supported using the ‘same indication’ criterion, stating that ‘comparing safety signals requires alignment in intended use’ (Table 2). However, the industry group highlighted regulatory variations, proposing that ‘comparable, if not identical, indications’ be considered instead.

Despite these differences, both groups agreed on the importance of safety report quality (Table 2). The academic group underscored that ‘safety reports should be prepared in compliance with the international guidelines and standards.’

For the accessibility of safety reports, the academic group highlighted transparency, stating that ‘the full safety report should be disclosed for reference.’ In contrast, the industry group focused on feasibility, emphasizing whether ‘domestic regulatory agencies can access foreign safety reports through international networks.’

Differences emerged regarding demographic similarity and the drug’s country of origin (Table 2). The academic group considered demographic characteristics, such as age and race, to be relevant, whereas the industry group argued that demographic similarity is rarely prioritized in practice, although referencing other Asian countries may be more applicable. In this context, demographic similarity was interpreted broadly to include factors such as genetic background, ethnicity, and epidemiological profiles that may influence drug response. Additionally, regarding the drug’s country of origin, the academic group supported its inclusion, reasoning that ‘regulators in the country of origin tend to act quickly based on comprehensive data.’ Conversely, the industry group questioned its relevance, arguing that ‘the concept is outdated, lacks a regulatory definition, and is difficult to specify due to the global nature of clinical trials.’

### Final proposed criteria based on FGI findings

A total of 11 criteria were selected from the FGIs and organized into two categories: Stage 1 and Stage 2 (Table 3). This study adopted the framework of the Australian Therapeutic Goods Administration (TGA) for identifying Comparable Overseas Regulators (CORs), as it is one of the few regulatory-endorsed models that explicitly outlines criteria for identifying comparable foreign regulators (Therapeutic Goods Administration, 2024). The COR framework consists of two stages: Stage 1 assesses regulator comparability, and Stage 2 applies product-specific criteria. Building on this structure, the criteria were organized into Stage 1 (country-level) and Stage 2 (drug-level) categories, incorporating additional factors highlighted by expert consensus, including the reliability and accessibility of safety reports. Definitions for each criterion are provided in Table 3.

**Table 3.** Definitions of the criteria presented in the survey.

Classification	Criteria	Explanation	Rationale of inclusion
Stage 1	Country-specific criteria		
1	Countries officially being referenced	The COR is officially referenced by multiple foreign regulatory agencies.	Reflects international regulatory trust and facilitates reliance-based decision-making.
2	Countries that monitor other countries	The COR actively monitors safety information from other countries.	Indicates active engagement in global pharmacovigilance and access to diverse safety information.
3	Countries with an advanced economy	The COR is a developed country with a high-income economy.	May serve as a proxy for infrastructure supporting regulatory and pharmacovigilance activities.
4	Countries with an advanced pharmaceutical industry	The COR is a developed country with a large pharmaceutical industry, typically measured by prescription drug sales.	Ensures the generation and management of high-quality and reliable safety data.
5	Countries with strong pharmaceutical safety management	The COR maintains an actively functioning pharmacovigilance system, as evidenced by the number of full-time staff in the safety department, active adverse event reporting, and ICH membership.	Ensures the generation and management of high-quality and reliable safety data.
6	Countries with a strong regulatory network	The COR has formal agreements with the MFDS, such as an MOU or a non-disclosure agreement.	Facilitates timely information exchange and access to otherwise restricted safety data.
Stage 2	Drug-specific criteria		
1	Comparable indication	The overseas safety report concerns indications equivalent to those of the drug under evaluation.	Enhances the clinical relevance and applicability of safety information.
2	Reliability of the report	Safety reports comply with the guidelines and standards adopted by the MFDS.	Directly affects the credibility and interpretability of safety evidence.
3	Accessibility of the report	The safety report is fully disclosed and accessible in its entirety for MFDS safety evaluations.	Determines the feasibility of incorporating the information into regulatory assessments.
4	Demographic similarity	The reference country's population possesses demographic or genetic characteristics similar to those of the Korean population, which may affect drug response.	Improves the generalizability of safety data to the target population.
5	Drug’s country of origin	The country where the headquarters of the company that developed the drug is located.	May reflect early access to safety data but has limited relevance in multinational development contexts.

COR, Comparable Overseas Regulator; ICH, International Council for Harmonization; MFDS, Ministry of Food and Drug Safety; MOU, Memorandum of Understanding.

### Relative weights derived from AHP

Of the 28 participants, 27 (96.4%) met the CR threshold and were included in the final analysis. The individual consistency ratios (CRs) for Stage 1 ranged from 0.020–0.149 (mean: 0.082; median: 0.076), while those for Stage 2 ranged from 0.009–0.132 (mean: 0.062; median: 0.054), indicating an overall acceptable level of consistency across respondents. The relative weights of the Stage 1 (country-specific) criteria derived from the AHP are presented in Table 4. The most highly weighted Stage 1 criterion was ‘countries officially being referenced’ (weight = 0.374), which was 1.46 and 2.77 times more important than ‘countries with a strong pharmaceutical safety management’ (0.256) and ‘countries with an advanced pharmaceutical industry’ (0.135), respectively. The next most important criterion was ‘countries that monitor other countries’ (0.104), which was approximately 3.60 times less important than the top-ranked criterion.

**Table 4.** Relative weights of country- and drug-specific criteria.

Criteria	Relative weight		
	All (n = 27)	Academic group (n = 11)	Industry group (n = 16)
Stage 1 (country-specific criteria)			
Countries officially being referenced	0.374	0.387	0.358
Countries with strong pharmaceutical safety management	0.256	0.197	0.302
Countries with an advanced pharmaceutical industry	0.135	0.136	0.133
Countries that monitor other countries	0.104	0.118	0.095
Countries with a strong regulatory network	0.070	0.073	0.066
Countries with an advanced economy	0.060	0.088	0.046
Subtotal	1.000	1.000	1.000
Stage 2 (drug-specific criteria)			
Reliability of the report	0.433	0.377	0.465
Accessibility of the report	0.181	0.220	0.154
Comparable indication	0.176	0.139	0.203
Demographic similarity	0.121	0.162	0.098
Drug’s country of origin	0.090	0.103	0.080
Subtotal	1.000	1.000	1.000

COR, Comparable Overseas Regulator; ICH, International Council for Harmonization; MFDS, Ministry of Food and Drug Safety; MOU, Memorandum of Understanding.

Subgroup analysis revealed consistent rankings between the academic and industry experts. However, minor differences were observed in the perceived relative importance of individual criteria. For instance, academic experts assigned 1.96 times more weight to the top criterion compared to the second-ranked criterion, whereas industry experts assigned relatively similar weights to the two (0.358 vs. 0.302). The least important criteria were ‘countries with a strong regulatory network’ (0.070) and ‘countries with an advanced economy’ (0.060). These criteria were weighted more than sixfold lower than the most highly weighted criterion.

For Stage 2 (drug-specific) criteria, ‘reliability of the report’ emerged as the most critical factor, with a weight of 0.433, which was 2.39 times greater than the weight assigned to ‘accessibility of the report’ (0.181) and 2.46 times greater than that of ‘comparable indication’ (0.176) (Table 4). Although the order of importance was consistent in the academic group, the industry group placed even greater emphasis on ‘reliability of the report’ (0.465) compared with the academic group (0.377). Among industry experts, ‘reliability of the report’ was 3.02 times more important than ‘accessibility of the report’ (0.154) and 2.29 times more important than ‘comparable indication’ (0.203). Both groups ranked ‘drug’s country of origin’ as the least important drug-specific criterion, assigning weights of 0.103 (academic) and 0.080 (industry), respectively, for a combined average weight of 0.090.

A sensitivity analysis using a stricter CR threshold of 0.10 confirmed the robustness of the results, as the relative ranking of criteria in both Stage 1 and Stage 2 remained unchanged (see Supplemental Table S4).

### Validity of the proposed framework

The validity and potential application of the proposed framework were evaluated through a second round of FGIs (Table 5). Experts from both academia and industry agreed that the relative importance values derived from the AHP, as presented in Table 4, were appropriate and reasonable. Specifically, participants emphasized that a country frequently referenced by other regulatory authorities represents the most critical factor for selecting a reference country. This observation aligns closely with the AHP results.

**Table 5.** Summary of expert opinions from the second round of FGIs on the proposed criteria and relative weights.

Category	Core Opinions
Opinions on the relative weights	(All) As confirmed by both the FGIs and the AHP, the frequency with which a country is officially referenced by others should be the most important selection criterion. (Industry) Ensuring the reliability and accessibility of safety reports is crucial, particularly from the perspective of practitioners.
Necessity of the criteria	(All) The criteria are valuable for monitoring overseas safety information, especially given the absence of formal guidelines for prioritizing countries in the PV systems.
Application of the framework in PV systems	(Industry) Implementing the proposed criteria may affect not only regulatory agencies but also the PV processes of pharmaceutical companies. A cautious approach is therefore recommended, with further stakeholder input before adoption.

In general, participants concurred that the prioritization of both country-specific and drug-specific criteria was appropriate. Industry professionals, in particular, highlighted the importance of accessibility of safety reports from a practitioner’s standpoint and expressed agreement with the weightings derived through the AHP. Experts from both groups acknowledged that the proposed criteria could facilitate the effective collection of overseas safety information, particularly in contexts where such data are extensive and heterogeneous.

However, regarding the practical application of the framework, industry experts raised concerns about its potential impact on existing PV systems. They recommended that public consultation and stakeholder engagement be conducted before full-scale implementation to ensure broader acceptance and minimize unintended consequences.

## Discussion

This study identified key criteria for selecting pharmaceutical safety information from foreign countries through expert interviews and analyzed the relative importance of each criterion using the AHP. The proposed framework is primarily intended to support regulatory prioritization, specifically in selecting which countries’ safety information should be referenced in decision-making. It is not designed for signal detection, but may facilitate risk assessment by enabling more structured use of existing safety information. In this context, the framework aligns with established pharmacovigilance processes, such as those outlined in ICH E2C (European Medicines Agency, 2014) and E2E (European Medicines Agency, 2004), by supporting the prioritization of external safety information that may inform benefit–risk evaluation and pharmacovigilance planning.

Among the Stage 1 (country-specific) criteria, ‘countries officially being referenced’ (0.374) emerged as the most important factor, underscoring the significance of recognition by other regulatory agencies in guiding domestic regulatory decisions. ‘Countries with excellent pharmaceutical safety management’ (0.256) ranked second, reflecting the value placed on robust regulatory frameworks. Subgroup analysis revealed notable differences between academic and industry participants. Specifically, ‘countries with a strong pharmaceutical safety management’ was weighted more heavily by the industry group (0.302) than by the academic group (0.197), indicating a 1.53-fold difference. This finding suggests that industry stakeholders may place greater emphasis on the regulatory capabilities of source countries when evaluating foreign safety information. By contrast, the criterion of ‘advanced economy’ was ranked very low, indicating that the overall size of a nation’s economy is not perceived as an important proxy for the reliability of pharmacovigilance information. Expert consultations suggested that what matters more for regulatory practice is the scale and sophistication of a country’s pharmaceutical industry, which more directly shapes the availability, quality, and timeliness of safety data.

For Stage 2 (drug-specific) criteria, the ‘reliability of the report’ was identified as the most critical factor by both the academic (0.377) and industry (0.465) groups, underscoring a shared prioritization of evidence quality. In contrast, ‘drug’s country of origin’ received the lowest weight from both groups (0.103 and 0.080, respectively), signaling a departure from relying on geographic origin as a proxy for data reliability and a shift toward more rigorous, evidence-based evaluations. This finding is consistent with expert opinions shared during the FGIs, where participants noted that although the country of origin may accumulate the most safety information, its relevance as a selection criterion is increasingly limited due to the growing prevalence of multinational clinical trials. This interpretation suggests that globalization of drug development has blurred the concept of a single ‘originating country,’ making the criterion less practical for regulatory referencing.

Previous studies have sought to improve international monitoring of pharmaceutical safety information. For example, one study on herbal PV synthesized expert interviews from 11 countries and identified key challenges, such as regulatory inconsistencies, difficulties in product identification, and limitations in adverse reaction reporting. The study proposed five strategic approaches, including increasing awareness, enhancing education, and strengthening legislative frameworks to improve the safety monitoring of herbal products (Ekhart et al., 2025). Another study proposed a structured approach for integrating drug safety evidence, consisting of three dimensions – evidence assessment, interpretation, and action – to facilitate more consistent regulatory decision-making (Hammad & Davies, 2025). While both studies provide valuable conceptual frameworks based on expert insights, the present study advances this work by quantifying the relative importance of each component, thereby offering a more structured and prioritized decision-making tool. This approach is particularly useful when regulatory decisions must be made under urgent conditions and with heterogeneous data. In such situations, the framework can support more timely and consistent decision-making by providing predefined criteria for prioritizing foreign safety information, thereby reducing reliance on ad hoc judgments under time constraints. Building on this, the final weighted criteria provide a basis for operational decision-making, including the systematic selection and prioritization of reference countries for safety information monitoring. Future work should focus on translating these criteria into practical implementation tools, such as scoring systems or decision thresholds, to support routine or emergency regulatory use.

This study has some limitations. First, the number of interview participants was relatively small, and as such, the findings may not be fully representative of the broader academic and pharmaceutical industry populations. However, the sample sizes align with established practices in qualitative and AHP research, where panels of approximately 15 experts are typically considered sufficient (Ishizaka & Labib, 2009). This study prioritized participant diversity and expertise of participants to enhance content validity. Despite the modest sample size, the inclusion of experts from diverse sectors – academia, PV, and regulatory affairs – supports the broader applicability of the framework to similar regulatory environments. Nevertheless, small panels may be more vulnerable to disproportionate influence by dominant perspectives or to unmeasured heterogeneity across experts, which could constrain the robustness of the derived weights. Although subgroup analyses by expert background (academic vs industry) showed consistent results, further stratification (e.g. regulatory affairs vs PV) was not performed due to the limited sample size, which may affect the stability of AHP-derived estimates. Second, the framework may unintentionally disadvantage emerging regulatory authorities, as criteria such as regulatory recognition and pharmacovigilance capacity tend to favor more established systems. However, the framework is designed to prioritize the reliability and relevance of safety information rather than the status of specific countries. Emerging regulatory authorities may still be considered if they demonstrate strengths in areas such as active safety monitoring, data accessibility, or increasing international recognition. Third, because the findings are based on the opinions of Korean experts, attention should be paid when generalizing our findings to other countries, which generate much of their own safety data (Kiguba et al., 2023). In particular, the results may reflect cultural and regulatory features unique to Korea, a country that relies heavily on imported pharmaceuticals and emphasizes referencing overseas safety data. This context may have shaped the relative importance assigned to certain criteria, and thus the framework’s applicability may differ in countries with stronger domestic pharmacovigilance infrastructures or alternative regulatory practices. Lastly, although the second-round FGIs were conducted to validate the AHP results, they relied on consensus-based discussions rather than independent assessment, and thus did not constitute a full form of external validation. Future studies should consider retrospective testing on regulatory cases or validation through independent expert panels.

Despite these limitations, the proposed framework has the potential to contribute to cross-border regulatory harmonization by providing consistent and transparent criteria for referencing international safety data. Such alignment could facilitate more coordinated post-marketing safety actions across countries with comparable PV needs, particularly those that rely on precedents set by overseas regulators. This relevance is especially pronounced for low- and middle-income countries (LMICs), where limited pharmacovigilance resources heighten dependence on foreign safety data (Olsson et al., 2015). Furthermore, future integration of the framework with artificial intelligence or machine learning technologies may enhance its scalability by enabling the automated filtering and classification of large volumes of safety information, while preserving expert-driven prioritization (Warner et al., 2025). In addition, given the dynamic nature of regulatory environments and evolving safety evidence, the framework may require periodic updating to maintain its relevance. For example, ICH E2C(R2) recommends regular reassessment at defined intervals – typically every 6–12 months in the initial phase, followed by longer intervals – alongside updates triggered by emerging safety information (European Medicines Agency, 2014). Accordingly, updates could follow a hybrid approach that combines regular review intervals with event-driven revisions in response to significant regulatory changes or newly identified safety signals.

To the best of current knowledge, this is the first study to develop a structured framework based on the AHP that specifically targets pharmaceutical experts for referencing foreign safety information in drug-specific regulatory contexts. The application of these criteria is expected to streamline the collection of relevant safety information and enhance the consistency and predictability of PV systems. By offering a structured, evidence-based approach to the selection of reference countries, this framework addresses a critical gap in the prioritization and interpretation of global safety information in regulatory settings. It provides a practical decision-support tool for regulators facing information overload, enabling more timely and consistent safety decisions.

## Supplementary Material

Supplemental Material

## Data Availability

The data for this manuscript (transcribed interviews and AHP responses) are not publicly available. Requests to access the datasets should be directed to the corresponding author and will be granted upon reasonable request.
