# Supplementary material for: Framework for identifying reference countries in drug safety evaluation: an application of the analytic hierarchy process
Source: J Pharm Policy Pract. 2026 Jun 1;19(1):2676817. doi: 10.1080/20523211.2026.2676817 (PMC13228168; doi:10.1080/20523211.2026.2676817)
Supplement: Supplemental Material [file JPPP_A_2676817_SM8535.docx]

**Appendix – supplementary material**

**AHP Questionnaire**

Supplementary Table 1. Questionnaire used in the AHP

| Through advisory meetings with academic experts and pharmaceutical company officials, we derived the criteria for selecting reference countries in drug safety evaluation, as shown in <Table 1>.  This survey aims to assess the relative importance of each criterion in Stage 1 and Stage 2.  After reviewing the explanation of each criterion, please indicate the relative importance of the two criteria presented at either end.  <Table> Criteria and explanations for selecting reference countries.   \| **The framework is applied in two stages:**   - Stage 1: Country-specific criteria to assess the adequacy of a COR - Stage 2: Drug-specific criteria relevant to the drug under evaluation  \| **Classification** \| **Criteria** \| **Explanation** \| \| --- \| --- \| --- \| \| Stage 1 \| Country-specific criteria \| \| \| 1 \| Countries officially referenced \| ∙ The COR is officially referenced by multiple foreign regulatory agencies. \| \| 2 \| Countries that monitor other countries \| ∙ The COR actively monitors safety information from other countries. \| \| 3 \| Countries with an advanced economy \| ∙ The COR is a developed country with a high-income economy. \| \| 4 \| Countries with an advanced pharmaceutical industry \| ∙ The COR is a developed country with a large pharmaceutical industry, typically measured by prescription drug sales. \| \| 5 \| Countries with strong pharmaceutical safety management \| ∙ The COR maintains an actively functioning pharmacovigilance system, as evidenced by the number of full-time staff in the safety department, active adverse event reporting, and ICH membership. \| \| 6 \| Countries with a strong regulatory network \| ∙ The COR has formal agreements with the MFDS, such as an MOU or a non-disclosure agreement. \| \| Stage 2 \| Drug-specific criteria \| \| \| 1 \| Comparable indication \| ∙ The overseas safety report concerns indications equivalent to those of the drug under evaluation. \| \| 2 \| Reliability of the report \| ∙ Safety reports comply with the guidelines and standards adopted by the MFDS. \| \| 3 \| Accessibility of the report \| ∙ The safety report is fully disclosed and accessible in its entirety for MFDS safety evaluations. \| \| 4 \| Demographic similarity \| ∙ The reference country's population possesses demographic or genetic characteristics similar to those of the Korean population, which may affect drug response. \| \| 5 \| Drug’s country of origin \| ∙ The country where the headquarters of the company that developed the drug is located. \| \| COR, Comparable Overseas Regulator; ICH, International Council for Harmonization; MFDS, Ministry of Food and Drug Safety; MOU, Memorandum of Understanding \| \| \| \| \| --- \| --- \| --- \| --- \| --- \| --- \| --- \| --- \| --- \| --- \| --- \| --- \| --- \| --- \| --- \| --- \| --- \| --- \| --- \| --- \| --- \| --- \| --- \| --- \| --- \| --- \| --- \| --- \| --- \| --- \| --- \| --- \| --- \| --- \| --- \| --- \| --- \| --- \| --- \| --- \| --- \| --- \| --- \| --- \| --- \| --- \|   **■ How to respond: If A is 5 times more important than B, mark 5. If B is 5 times more important than A, mark 1/5**   \| **criterion** \| Absolute importance \| Very strong importance \| strong importance \| Moderate importance \| Equal \| Moderate importance \| strong importance \| Very strong importance \| Absolute importance \| **criterion** \| \| --- \| --- \| --- \| --- \| --- \| --- \| --- \| --- \| --- \| --- \| --- \| \| **A** \| 9 \| 7 \| 5 \| 3 \| 1 \| 1/3 \| 1/5 \| 1/7 \| 1/9 \| **B** \|   **1. The following are criteria for determining whether a country's regulatory agency meets the requirements for selection as a reference country by the Ministry of Food and Drug Safety. Please indicate the relative importance of each criterion. (√)**   \|  \| criterion \| Absolute importance \| Very strong importance \| strong importance \| Moderate importance \| Equal \| Moderate importance \| strong importance \| Very strong importance \| Absolute importance \| criterion \| \| --- \| --- \| --- \| --- \| --- \| --- \| --- \| --- \| --- \| --- \| --- \| --- \| \| 1) \| Countries officially referenced \| 9 \| 7 \| 5 \| 3 \| 1 \| 1/3 \| 1/5 \| 1/7 \| 1/9 \| Countries that monitor other countries \| \| 2) \| Countries officially referenced \| 9 \| 7 \| 5 \| 3 \| 1 \| 1/3 \| 1/5 \| 1/7 \| 1/9 \| Countries with an advanced economy \| \| 3) \| Countries officially referenced \| 9 \| 7 \| 5 \| 3 \| 1 \| 1/3 \| 1/5 \| 1/7 \| 1/9 \| Countries with an advanced pharmaceutical industry \| \| 4) \| Countries officially referenced \| 9 \| 7 \| 5 \| 3 \| 1 \| 1/3 \| 1/5 \| 1/7 \| 1/9 \| Countries with strong pharmaceutical safety management \| \| 5) \| Countries officially referenced \| 9 \| 7 \| 5 \| 3 \| 1 \| 1/3 \| 1/5 \| 1/7 \| 1/9 \| Countries with a strong regulatory network \| \| 6) \| Countries that monitor other countries \| 9 \| 7 \| 5 \| 3 \| 1 \| 1/3 \| 1/5 \| 1/7 \| 1/9 \| Countries with an advanced economy \| \| 7) \| Countries that monitor other countries \| 9 \| 7 \| 5 \| 3 \| 1 \| 1/3 \| 1/5 \| 1/7 \| 1/9 \| Countries with an advanced pharmaceutical industry \| \| 8) \| Countries that monitor other countries \| 9 \| 7 \| 5 \| 3 \| 1 \| 1/3 \| 1/5 \| 1/7 \| 1/9 \| Countries with strong pharmaceutical safety management \| \| 9) \| Countries that monitor other countries \| 9 \| 7 \| 5 \| 3 \| 1 \| 1/3 \| 1/5 \| 1/7 \| 1/9 \| Countries with a strong regulatory network \| \| 10) \| Countries with an advanced economy \| 9 \| 7 \| 5 \| 3 \| 1 \| 1/3 \| 1/5 \| 1/7 \| 1/9 \| Countries with an advanced pharmaceutical industry \| \| 11) \| Countries with an advanced economy \| 9 \| 7 \| 5 \| 3 \| 1 \| 1/3 \| 1/5 \| 1/7 \| 1/9 \| Countries with strong pharmaceutical safety management \| \| 12) \| Countries with an advanced economy \| 9 \| 7 \| 5 \| 3 \| 1 \| 1/3 \| 1/5 \| 1/7 \| 1/9 \| Countries with a strong network \| \| 13) \| Countries with an advanced pharmaceutical industry \| 9 \| 7 \| 5 \| 3 \| 1 \| 1/3 \| 1/5 \| 1/7 \| 1/9 \| Countries with strong pharmaceutical safety management \| \| 14) \| Countries with an advanced pharmaceutical industry \| 9 \| 7 \| 5 \| 3 \| 1 \| 1/3 \| 1/5 \| 1/7 \| 1/9 \| Countries with a strong regulatory network \| \| 15) \| Countries with strong pharmaceutical safety management \| 9 \| 7 \| 5 \| 3 \| 1 \| 1/3 \| 1/5 \| 1/7 \| 1/9 \| Countries with a strong regulatory network \|   **2. The following are criteria for determining whether regulations have been applied with consideration of the characteristics of a drug subject to safety measures in a particular country. Please indicate the relative importance of each criterion. (√)**   \|  \| criterion \| Absolute importance \| Very strong importance \| strong importance \| Moderate importance \| Equal \| Moderate importance \| strong importance \| Very strong importance \| Absolute importance \| criterion \| \| --- \| --- \| --- \| --- \| --- \| --- \| --- \| --- \| --- \| --- \| --- \| --- \| \| 1) \| Comparable indication \| 9 \| 7 \| 5 \| 3 \| 1 \| 1/3 \| 1/5 \| 1/7 \| 1/9 \| Reliability of the report \| \| 2) \| Comparable indication \| 9 \| 7 \| 5 \| 3 \| 1 \| 1/3 \| 1/5 \| 1/7 \| 1/9 \| Accessibility of the report \| \| 3) \| Comparable indication \| 9 \| 7 \| 5 \| 3 \| 1 \| 1/3 \| 1/5 \| 1/7 \| 1/9 \| Demographic similarity \| \| 4) \| Comparable indication \| 9 \| 7 \| 5 \| 3 \| 1 \| 1/3 \| 1/5 \| 1/7 \| 1/9 \| Drug’s country of origin \| \| 5) \| Reliability of the report \| 9 \| 7 \| 5 \| 3 \| 1 \| 1/3 \| 1/5 \| 1/7 \| 1/9 \| Accessibility of the report \| \| 6) \| Reliability of the report \| 9 \| 7 \| 5 \| 3 \| 1 \| 1/3 \| 1/5 \| 1/7 \| 1/9 \| Demographic similarity \| \| 7) \| Reliability of the report \| 9 \| 7 \| 5 \| 3 \| 1 \| 1/3 \| 1/5 \| 1/7 \| 1/9 \| Drug’s country of origin \| \| 8) \| Accessibility of the report \| 9 \| 7 \| 5 \| 3 \| 1 \| 1/3 \| 1/5 \| 1/7 \| 1/9 \| Demographic similarity \| \| 9) \| Accessibility of the report \| 9 \| 7 \| 5 \| 3 \| 1 \| 1/3 \| 1/5 \| 1/7 \| 1/9 \| Drug’s original country of development \| \| 10) \| Demographic similarity \| 9 \| 7 \| 5 \| 3 \| 1 \| 1/3 \| 1/5 \| 1/7 \| 1/9 \| Drug’s country of origin \|   Thank you for your participation. |
| --- | --- | --- | --- | --- | --- | --- | --- | --- | --- | --- | --- | --- | --- | --- | --- | --- | --- | --- | --- | --- | --- | --- | --- | --- | --- | --- | --- | --- | --- | --- | --- | --- | --- | --- | --- | --- | --- | --- | --- | --- | --- | --- | --- | --- | --- | --- | --- | --- | --- | --- | --- | --- | --- | --- | --- | --- | --- | --- | --- | --- | --- | --- | --- | --- | --- | --- | --- | --- | --- | --- | --- | --- | --- | --- | --- | --- | --- | --- | --- | --- | --- | --- | --- | --- | --- | --- | --- | --- | --- | --- | --- | --- | --- | --- | --- | --- | --- | --- | --- | --- | --- | --- | --- | --- | --- | --- | --- | --- | --- | --- | --- | --- | --- | --- | --- | --- | --- | --- | --- | --- | --- | --- | --- | --- | --- | --- | --- | --- | --- | --- | --- | --- | --- | --- | --- | --- | --- | --- | --- | --- | --- | --- | --- | --- | --- | --- | --- | --- | --- | --- | --- | --- | --- | --- | --- | --- | --- | --- | --- | --- | --- | --- | --- | --- | --- | --- | --- | --- | --- | --- | --- | --- | --- | --- | --- | --- | --- | --- | --- | --- | --- | --- | --- | --- | --- | --- | --- | --- | --- | --- | --- | --- | --- | --- | --- | --- | --- | --- | --- | --- | --- | --- | --- | --- | --- | --- | --- | --- | --- | --- | --- | --- | --- | --- | --- | --- | --- | --- | --- | --- | --- | --- | --- | --- | --- | --- | --- | --- | --- | --- | --- | --- | --- | --- | --- | --- | --- | --- | --- | --- | --- | --- | --- | --- | --- | --- | --- | --- | --- | --- | --- | --- | --- | --- | --- | --- | --- | --- | --- | --- | --- | --- | --- | --- | --- | --- | --- | --- | --- | --- | --- | --- | --- | --- | --- | --- | --- | --- | --- | --- | --- | --- | --- | --- | --- | --- | --- | --- | --- | --- | --- | --- | --- | --- | --- | --- | --- | --- | --- | --- | --- | --- | --- | --- | --- | --- | --- | --- | --- | --- | --- | --- | --- | --- | --- | --- | --- | --- | --- | --- | --- | --- | --- | --- | --- | --- | --- | --- | --- | --- | --- | --- | --- | --- | --- | --- | --- | --- | --- | --- | --- | --- | --- | --- | --- | --- | --- | --- | --- | --- | --- | --- | --- | --- | --- | --- | --- | --- | --- | --- | --- | --- | --- | --- | --- | --- | --- | --- | --- | --- | --- | --- | --- | --- | --- | --- | --- | --- | --- | --- | --- | --- | --- | --- | --- | --- | --- | --- | --- | --- | --- | --- |

**Paired Comparison Matrix**

Below are the supplementary tables showing the full paired comparison matrix for stage 1 and stage 2 criteria.

Supplementary Table 2. Full comparison matrix for stage 1 criteria

|  | Criteria 1 | Criteria 2 | Criteria 3 | Criteria 4 | Criteria 5 | Criteria 6 |
| --- | --- | --- | --- | --- | --- | --- |
| Criteria 1 | 1.000 | 4.200 | 5.836 | 3.060 | 1.429 | 4.485 |
| Criteria 2 | 0.238 | 1.000 | 1.763 | 0.766 | 0.419 | 1.693 |
| Criteria 3 | 0.171 | 0.567 | 1.000 | 0.420 | 0.274 | 0.766 |
| Criteria 4 | 0.327 | 1.305 | 2.382 | 1.000 | 0.523 | 2.069 |
| Criteria 5 | 0.700 | 2.389 | 3.647 | 1.913 | 1.000 | 4.282 |
| Criteria 6 | 0.223 | 0.591 | 1.306 | 0.483 | 0.234 | 1.000 |

Supplementary Table 3. Full comparison matrix for stage 2 criteria

|  | Criteria 1 | Criteria 2 | Criteria 3 | Criteria 4 | Criteria 5 |
| --- | --- | --- | --- | --- | --- |
| Criteria 1 | 1.000 | 0.417 | 0.969 | 1.546 | 1.825 |
| Criteria 2 | 2.396 | 1.000 | 2.751 | 3.788 | 4.095 |
| Criteria 3 | 1.032 | 0.363 | 1.000 | 1.522 | 2.252 |
| Criteria 4 | 0.647 | 0.264 | 0.657 | 1.000 | 1.565 |
| Criteria 5 | 0.548 | 0.244 | 0.444 | 0.639 | 1.000 |

**Sensitivity Analysis**

Below is a supplementary table showing the weighted results when the consistency ratio is set to 0.10. Responses with a consistency ratio exceeding 0.10 were excluded from the results analysis.

Supplementary Table 4. Relative weight result of the sensitivity analysis when the consistency ratio is set to 0.1

| Stage 1 (Country-specific criteria) | Relative weight | | |
| --- | --- | --- | --- |
|  | All  (n=17) | Academic group  (n=8) | Industry group  (n=9) |
| Countries officially referenced | 0.406 | 0.387 | 0.408 |
| Countries with strong pharmaceutical safety management | 0.229 | 0.177 | 0.279 |
| Countries with an advanced pharmaceutical industry | 0.120 | 0.153 | 0.092 |
| Countries that monitor other countries | 0.115 | 0.103 | 0.123 |
| Countries with a strong regulatory network | 0.066 | 0.074 | 0.057 |
| Countries with an advanced economy | 0.064 | 0.105 | 0.041 |
| Subtotal | 1.000 | 1.000 | 1.000 |
| Stage 2 (Drug-specific criteria) | Relative weight | | |
|  | All  (n=21) | Academic group  (n=8) | Industry group  (n=13) |
| Reliability of the report | 0.443 | 0.392 | 0.459 |
| Accessibility of the report | 0.184 | 0.243 | 0.149 |
| Comparable indication | 0.163 | 0.096 | 0.218 |
| Demographic similarity | 0.120 | 0.167 | 0.094 |
| Drug’s country of origin | 0.090 | 0.102 | 0.080 |
| Subtotal | 1.000 | 1.000 | 1.000 |


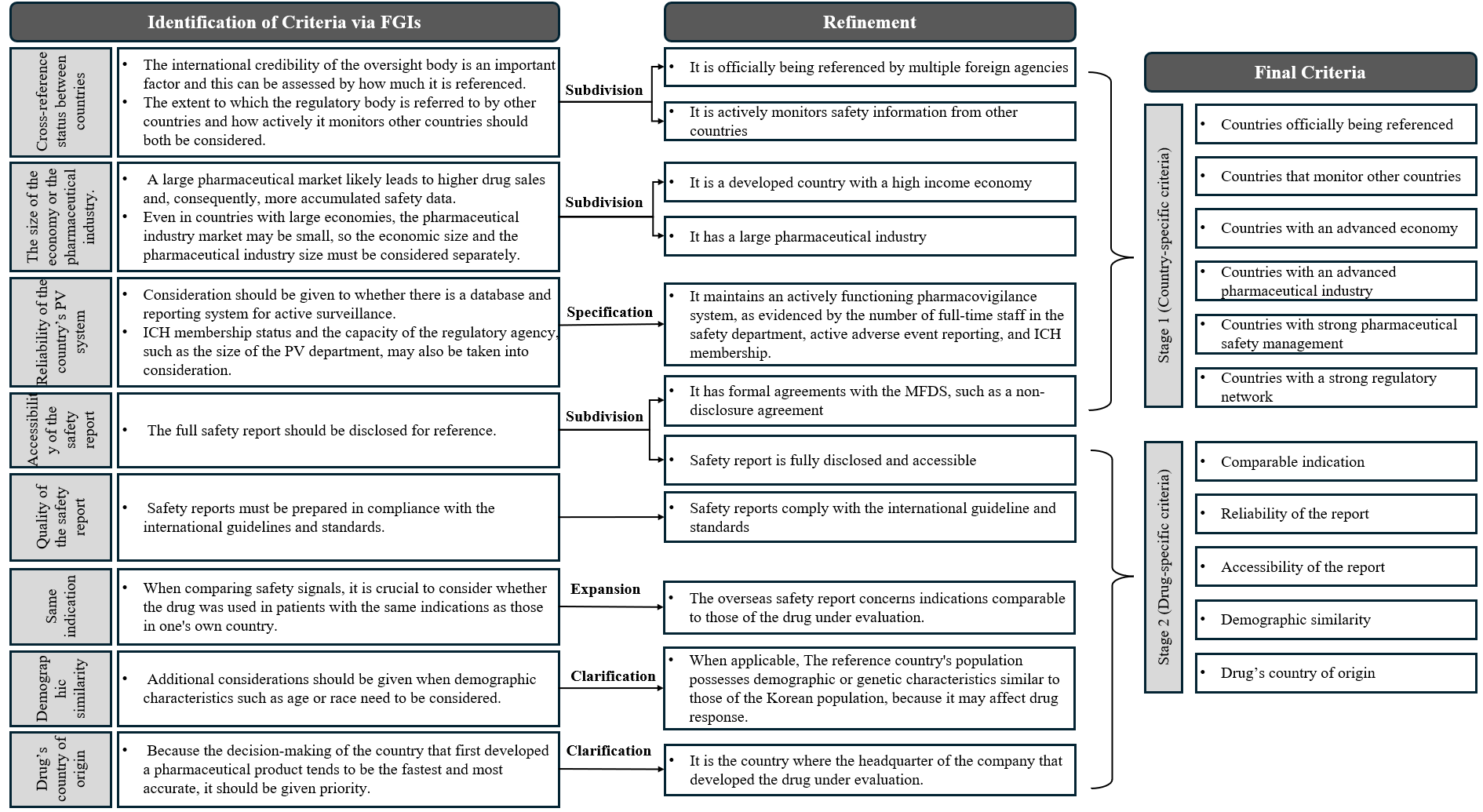


Supplementary figure 1. Flow diagram of criteria identification and refinement based on FGIs

**Ethics Statement**

The FGIs conducted in this study were not subject to ethics review, as no personal information was collected and participants shared their views in a professional capacity, not as private individuals. Furthermore, since the study was commissioned as part of a government policy initiative, it was exempt from Institutional Review Board (IRB) review under the Bioethics and Safety Act of the Republic of Korea (Korea Law Information Center, 2023).

Korea Law Information Center. (2023, December 5). *Bioethics and Safety Act*. https://www.law.go.kr/%EB%B2%95%EB%A0%B9/%EC%83%9D%EB%AA%85%EC%9C%A4%EB%A6%AC%EB%B0%8F%EC%95%88%EC%A0%84%EC%97%90%EA%B4%80%ED%95%9C%EB%B2%95%EB%A5%A0%EC%8B%9C%ED%96%89%EA%B7%9C%EC%B9%99
